# Supplementary material for: TRAF3 gene regulates macrophage migration and activation by lung epithelial cells infected with Aspergillus fumigatus
Source: Microbiol Spectr. 2023 Nov 29;12(1):e02699-23. doi: 10.1128/spectrum.02699-23 (PMC10783100; doi:10.1128/spectrum.02699-23)
Supplement: Fig. S1 and S2 — Fig. S1: Role of TRAF3 in the interaction between lung epithelial cells and macrophages in A. fumigatus infection. Fig. S2: Effect of TRAF3 to the viability and cell membrane integrity of lung epithelial cells infected with A. fumigatus. [file spectrum.02699-23-s0001.docx]

**SUPPLEMENTARY METHODS**

**MTT assay**

A549 cells were seeded at 1 × 10^4^ cells/well into 96-well plates (Corning, Costar, New York, USA) and cultivated overnight in a cell incubator at 37°C with 5% CO_2_. *A. fumigatus* spores were co-cultured with A549 cells at MOI = 10:1 for 6 h. Add 20 μL MTT reagent (SIGMA, USA) to the wells and incubate at 37°C for 2 h. Discard the liquid in the wells and add 200 μL DMSO (SIGMA, USA) to each well and mix thoroughly. The absorbance was detected at 490 nm using a microplate spectrophotometer (Gene Company Limited, Shanghai, China).

**LDH release assay**

A549 cells were seeded at 1 × 10^4^ cells/well into 96-well plates and cultivated overnight in a cell incubator at 37°C with 5% CO_2_. *A. fumigatus* spores were co-cultured with A549 cells at MOI = 10:1 for 6 h. The cell culture supernatants of different groups were collected and centrifuged at 3000 rpm for 5 min. Afterward,120 μL of cell culture supernatant was added to a new 96-well plate. Add 60 μL of LDH detection working solution(Beyotime Biotechnology, China) to each well, and incubate for 30 min at room temperature and away from light. The absorbance was detected at 490 nm using a microplate spectrophotometer.


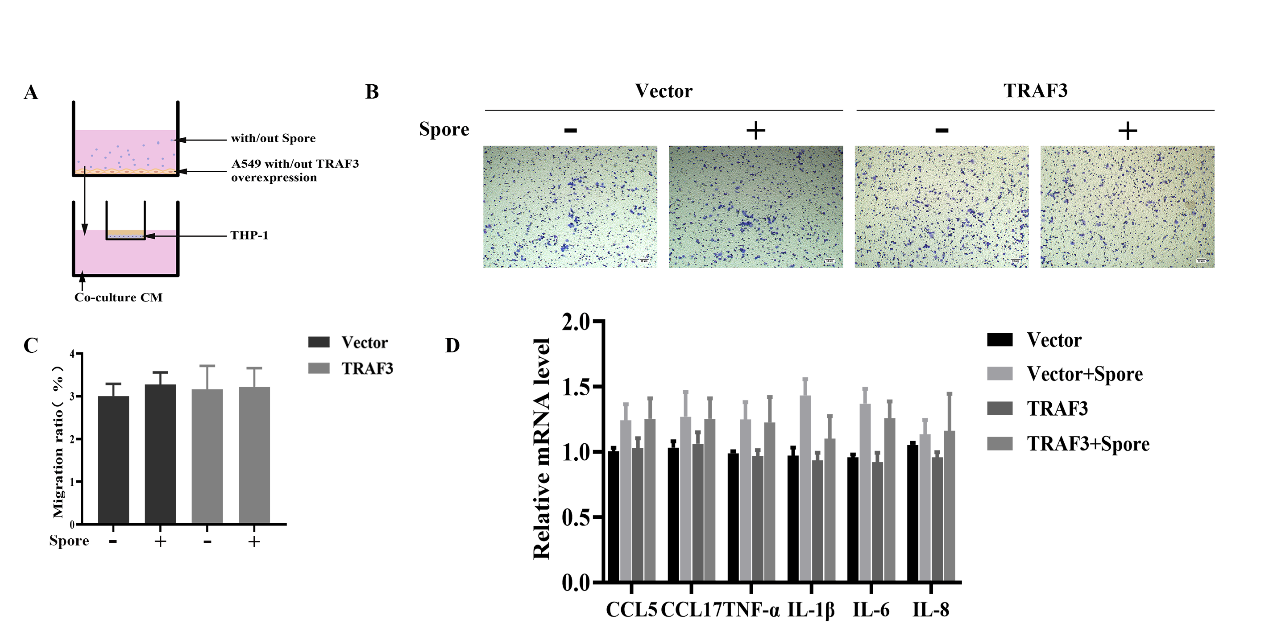


Supplementary Figure 1. Role of TRAF3 in the interaction between lung epithelial cells and macrophages in *A. fumigatus* infection （A）Schematic diagram of *A. fumigatus*-lung epithelial cell co-culture supernatant-stimulated macrophages. (B, C) Macrophages that migrated to the lower chamber were fixed with 4% paraformaldehyde, stained with Giemsa stain and counted. (D) The expression of cytokines in the macrophages in the upper Transwell chamber was analyzed by quantitative real-time PCR.


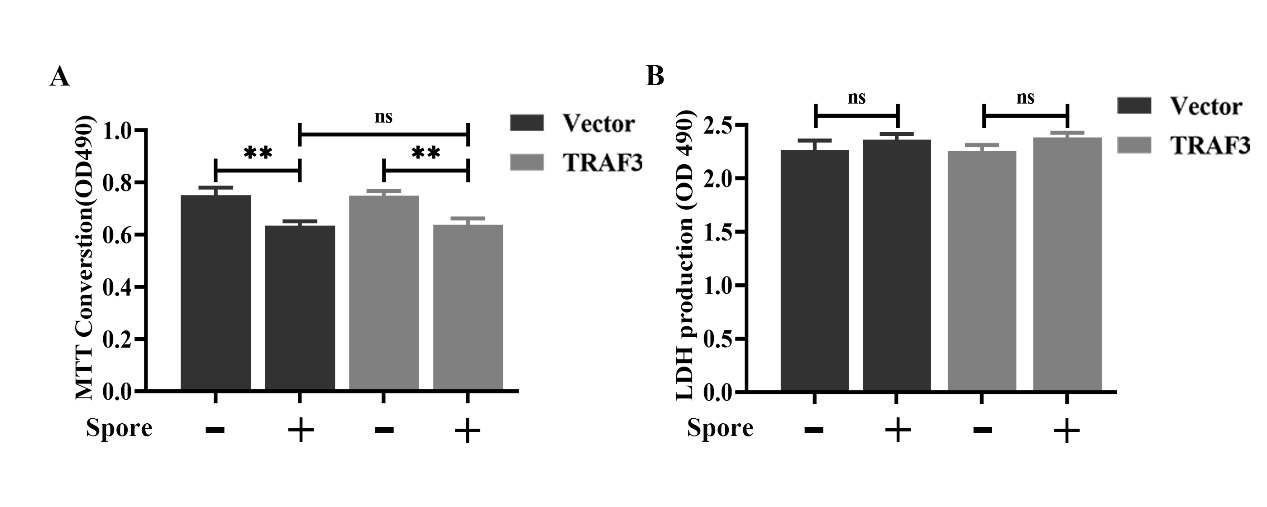
Supplementary Figure 2. Effect of TRAF3 to the viability and cell membrane integrity of lung epithelial cells infected with *A. fumigatus* (A) Viability of A549 cells was detected by MTT conversion. (B) Detection of A549 cell membrane integrity by LDH release.
